# Low Dietary Variety and Diabetes Mellitus Are Associated with Frailty among Community-Dwelling Older Japanese Adults: A Cross-Sectional Study

**DOI:** 10.3390/nu13020641

**Published:** 2021-02-16

**Authors:** Misato Hayakawa, Keiko Motokawa, Yurie Mikami, Kaori Yamamoto, Maki Shirobe, Ayako Edahiro, Masanori Iwasaki, Yuki Ohara, Yutaka Watanabe, Hisashi Kawai, Motonaga Kojima, Shuichi Obuchi, Yoshinori Fujiwara, Hunkyung Kim, Kazushige Ihara, Hiroki Inagaki, Shoji Shinkai, Shuichi Awata, Atsushi Araki, Hirohiko Hirano

**Affiliations:** 1 Research Team for Promoting Independence and Mental Health, Tokyo Metropolitan Institute of Gerontology, 35-2 Sakae-cho, Itabashi-ku, Tokyo 173-0015, Japan; mh24@tmig.or.jp (M.H.); ega0dm@gmail.com (Y.M.); yamamoto0915093@gmail.com (K.Y.); aedahiro514@gmail.com (A.E.); iwasaki@tmig.or.jp (M.I.); yohara@tmig.or.jp (Y.O.); kimhk@centm.center.tmig.or.jp (H.K.); inagaki@tmig.or.jp (H.I.); awata@tmig.or.jp (S.A.); h-hiro@gd5.so-net.ne.jp (H.H.); 2 The Tokyo Metropolitan Support Center for Preventative Long-term and Frail Elderly Care, Tokyo Metropolitan Institute of Gerontology, 25-8 Oyamakanai-cho, Itabashi-ku, Tokyo 173-0024, Japan; mshirobe@tmig.or.jp (M.S.); fujiwayo@tmig.or.jp (Y.F.); 3 Gerodontology, Department of Oral Health Science, Faculty of Dental Medicine, Hokkaido University, Kita 8, Nishi 5, Kita-ku, Sapporo, Hokkaido 060-0808, Japan; ywata@den.hokudai.ac.jp; 4 Research Team for Human Care, Tokyo Metropolitan Institute of Gerontology, 35-2 Sakae-cho, Itabashi-ku, Tokyo 173-0015, Japan; hkawai@tmig.or.jp (H.K.); obuchipc@tmig.or.jp (S.O.); 5 Department of Physical Therapy, University of Tokyo Health Sciences, 4-11 Ohiai, Tama-shi, Tokyo 206-0033, Japan; m-kojima@u-ths.ac.jp; 6 Research Team for Social Participation and Community Health, Tokyo Metropolitan Institute of Gerontology, 35-2 Sakae-cho, Itabashi-ku, Tokyo 173-0015, Japan; 7 Graduate School of Medicine, Hirosaki University, 5 Zaifu-cho, Hirosaki-shi, Aomori 036-8562, Japan; ihara@hirosaki-u.ac.jp; 8 Graduate School of Nutrition and Health Science, Kagawa Nutrition University, 3-9-21 Chiyoda, Sakado-shi, Saitama 350-0288, Japan; sshinkai1955@yahoo.co.jp; 9 Department of Diabetes, Metabolism, and Endocrinology, Tokyo Metropolitan Geriatric Hospital, 35-2 Sakae-cho, Itabashi-ku, Tokyo 173-0015, Japan; aaraki@tmghig.jp

**Keywords:** frailty, diabetes, Dietary Variety Score

## Abstract

The association between dietary diversity and frailty remains unknown in older people. We evaluated whether a limited dietary variety is associated with frailty in older adults with diabetes mellitus (DM). This cross-sectional investigation included 1357 adults (median age: 77 years, women: 61.3%). DM was determined by self-reporting, the Dietary Variety Score (DVS) was used to evaluate dietary variety, and the revised Japanese version of the Cardiovascular Health Study criteria evaluated frailty. Participants were divided into 4 groups: no DM/high DVS (non-DMHV), no DM/low DVS (non-DMLV), DM/high DVS (DMHV), and DM/low DVS (DMLV). The prevalence of frailty in each group was 3.6%, 6.7%, 6.7%, and 12.2%. After adjusting for covariates, logistic regression analysis revealed the highest odds ratio (OR) of frailty in the DMLV (non-DMLV, OR = 2.18 (95% confidence interval (CI): 1.25–3.83); DMHV, OR = 1.87 (95% CI: 0.63–5.52); DML, OR = 5.03 (95% CI: 2.05–12.35)). Another logistic regression analysis revealed that a low DVS and DM were independently associated with frailty. Both a low dietary variety and DM were independently related to frailty in older people and the combination increased the prevalence of frailty. These findings suggest that high dietary variety could be important for the prevention of frailty in people with DM.

## 1. Introduction

As the population aging rate continues to increase, it is of the utmost importance to prevent disability and extend healthy life expectancy. Frailty is defined as being vulnerable to falls, disability, hospitalization, and mortality after a stressor event due to the age-related loss of functional reserve. Appropriate interventions, such as diet and exercise, can improve the condition of people with frailty, as it is reversible [1]. National prevalence of frailty in the older Japanese population is reported to be 8.7% [2]. Hence, there is a growing interest in the early recognition and prevention of frailty.

According to diabetes surveys and national health/nutrition surveys in Japan, the incidence of people with strongly suspected diabetes mellitus (DM) has constantly increased over the past years: 6.9 million in 1997, 8.9 million in 2007, and 10 million in 2016 [3,4,5]. The prevalence of diabetes is higher among older adults. Increased insulin resistance and reduced insulin secretion lead to reduced glucose tolerance, rendering older adults more susceptible to DM [6,7]. Studies have reported that DM is associated with frailty [8,9]. Although hyperglycemia [10,11], hypoglycemia [12], low hemoglobin A1c (HbA1c) [13], hyperlipidemia [11], abdominal obesity [11], macrovascular complications [14], and low levels of physical activity [11] are associated with frailty, few studies have been conducted on the role of nutrition in the increased risk of frailty in older individuals with DM. Malnutrition associated with comorbidities in old age and the overly strict dietary management of DM may lead to frailty.

A decrease in the intake of total energy and specific nutrients, such as protein and vitamins A and B, is reported to be associated with frailty [15,16,17,18], whereas Mediterranean and other healthy dietary patterns are associated with reduced frailty [19,20]. However, these dietary patterns are complex and may vary according to ethnic region. Dietary diversity is determined by assessing the number of food types that are consumed at least once per week—this is useful in the simple assessment of a balanced diet. Diversifying food intake may help older people consume a sufficient amount of energy and nutrients and avoid skipping meals. We previously reported that the degree of dietary variety was associated with the degree of frailty in the older Japanese population [21]. However, to the best of our knowledge, no studies have examined the combination of a low dietary variety and DM as a factor associated with frailty. Therefore, we postulated that people with DM who consume a low dietary variety are likely to have frailty. The study aimed to shed light on the relationship of frailty with dietary variety and DM in the older population.

## 2. Materials and Methods

### 2.1. Study Design and Participants

**Figure 1:**
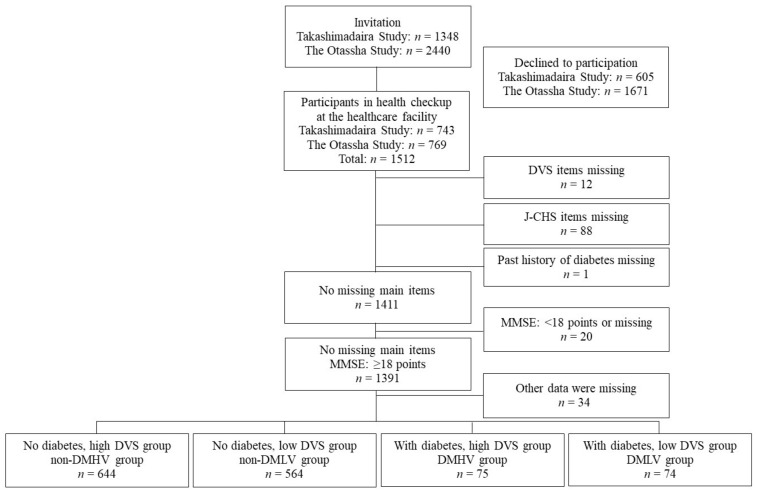
Flow chart showing details of participant recruitment. The current cross-sectional investigation used integrated data from two cohort studies: the Takashimadaira Study and the Otassha Study. In 2018, the Takashimadaira Study included community-dwelling older adults aged ≥ 72 years and the Otassha Study included community-dwelling older adults between the ages of 65 and 84.

This study used pooled data from the Takashimadaira Study and the Otassha Study, which are cohort studies concerning older community members of the Tokyo metropolitan area of Japan. The designs and protocols of the two studies have been described in detail elsewhere [22,23,24]. The Takashimadaira Study was initiated in 2016 and targeted adults aged ≥ 70 years living at Takashimadaira, Itabashi ward, Tokyo, Japan. We used data obtained at a 2-year follow-up survey conducted at a healthcare facility in 2018. The Otassha Study is a health checkup that has been conducted annually since 2011 for local residents between the ages of 65 and 84 at Itabashi ward, Tokyo, Japan. Since 2012, we have recruited past participants and new 65-year-old ones. We used data obtained from a follow-up survey conducted at a healthcare facility in 2018. In total, 1512 individuals (743 from the Takashimadaira Study and 769 from the Otassha Study) participated in the survey. Of these, the following candidates were excluded: 12 for whom meal evaluations were incomplete, 88 for whom frailty evaluation items were missing, and 1 for whom the DM evaluation was incomplete. With reference to earlier studies by Fried et al. and Motokawa et al., an additional 20 people were excluded for whom the evaluation of cognitive function was incomplete or who had reduced cognitive function (Mini-Mental State Examination (MMSE) score: <18 points) [1,21]. Moreover, another 34 people for whom other data were missing were also excluded. The remaining 1357 people were included in this study. Detailed methods of participant recruitment are summarized in Figure 1.

This study was approved by the research ethics committee of the Tokyo Metropolitan Geriatric Hospital and Institute of Gerontology (approval numbers for the Takashimadaira Study: 28–31, 30–33; approval numbers for The Otassha Study: 48 for 2011 and 16 for 2018). Written informed consent was obtained from all participants.

### 2.2. Evaluation of DM and Comorbidity

A well-experienced nurse interviewed participants to determine if they had a medical history of DM. A medical history of comorbidities (hypertension, hyperlipidemia, stroke, heart disease, osteoporosis, spinal canal stenosis, malignant neoplasm, and depression) and knee pain was also taken.

### 2.3. Evaluation of Dietary Variety Score

At healthcare facility, interviews about Dietary Variety Score (DVS) were conducted by trained investigators using the paper-based questionnaires. The DVS developed by Kumagai et al. [25] was used for the evaluation of variety in the participants’ everyday diet. The DVS includes the following 10 food categories: meat, seafood, eggs, soy products, milk, green and yellow vegetables, seaweed, potatoes, fruit, and fats and oils. For each category, a score of 1 is allocated if the participants’ answers of “eating almost every day,” and a score of 0 is allocated for all the remaining answers. The total score can range from 0 to 10. If even one food was defective, the total score could not be calculated, so it was excluded. In this study, the median was set as the reference. Scores from 0 to 4 were defined as low scores, and those > 5 as high scores. Participants were allocated to either the low or high score group. The DVS has been widely used in nutritional epidemiological studies of older adults. In addition, its validity was tested against the 3-day weighed food record method. Individuals with a higher DVS have previously reported lower grain energy proportions and significantly higher intakes of protein and micronutrients [26].

### 2.4. Assessment of Frailty

This study used the revised Japanese version of the Cardiovascular Health Study (revised J-CHS) for the evaluation of frailty [27,28]. The following 5 items were evaluated: weight loss, muscle weakness, fatigue, reduced walking speed, and reduced physical activity. Grip strength and walking speed were objectively measured by trained investigators in a visitor-based survey. Men with a grip strength of less than 28 kg and women with a grip strength of less than 18 kg were considered to have muscle weakness, while those walking at a speed slower than 1.0 m/s were considered to have reduced walking speed. Participants who answered “yes” to a question that asked them whether or not they lost 2 kg or more in the past 6 months were considered to have lost weight, participants who answered “yes” to a question that asked them whether or not they felt tired for no reason in the last two weeks were considered to have fatigue, and those who answered “no” to questions that asked them whether or not they engaged in light exercise or gymnastics and whether or not they regularly engaged in exercise or sports were considered to have reduced physical activity. Participants to whom three or more items applied were considered frail, those to whom 1–2 items applied were considered pre-frailty, and those to whom no items applied were considered robust.

### 2.5. Other Assessments

The following items were evaluated: sex, age, body mass index (BMI), the number of household members, alcohol consumption (yes: currently drinks alcohol, no: does not drink alcohol or used to drink alcohol), and smoking (yes: currently smokes, no: does not smoke or used to smoke). Serum levels of creatinine, albumin, and HbA1c were measured using conventional laboratory methods.

### 2.6. Statistical Analysis

Participants were divided into the following 4 groups based on the results of the DM and dietary evaluations: the no DM/high DVS (non-DMHV) group, no DM/low DVS (non-DMLV) group, the DM/high DVS (DMHV) group, and the DM/low DVS (DMLV) group. The Kruskal–Wallis test for continuous values and chi-square test for categorical variables were used to assess the differences between these 4 groups. A chi-square test was also performed for the presence of DM and DVS scores. Results are expressed as median (interquartile range (IQR)) and numbers of participants (%). Binomial logistic regression analysis was performed to examine the independent relationship between the above-defined 4 groups and frailty in the crude model. In the adjusted model 1, demographic variables (age, sex, and BMI) were adjusted. In the adjusted model 2, variables about lifestyles, comorbidity, and laboratory findings, which significantly associated with DM/DVS groups in univariate analysis, were further adjusted: alcohol consumption, hypertension, hyperlipidemia, heart disease, osteoporosis, the presence of knee pain, and serum creatinine. Another logistic regression analysis using the DVS and DM as independent variables was conducted to examine whether dietary variety and DM are independently associated with frailty. SPSS Statistics ver. 25 (IBM Corporation, Tokyo, Japan) was used for all statistical analyses. *p*-values were set at 5%.

## 3. Results

The median (IQR) of the age of participants was 77 (72–81) years. Approximately 61.3% (*n* = 832) were women. Table 1 shows the characteristics of the 4 groups classified by the presence or absence of DM and a high or low DVS. The number of participants in each group was as follows: non-DMHV (*n* = 644, 47.5%), non-DMLV (*n* = 564, 41.6%), DMHV *(n* = 75, 5.5%), and DMLV (*n* = 74, 5.5%). The median BMI, DVS, serum creatinine, and HbA1c, and the prevalence of sex, alcohol consumption, a past history of hypertension, heart disease, hyperlipidemia, and osteoporosis were significantly different among the 4 groups. There were significant differences in the frequency of all foods consumed daily between the high DVS and low DVS groups in participants with and without DM (Table S1).

Table 2 shows the prevalence of robust, pre-frailty, and frailty in the 4 groups. It was found that the combination of DM and a high dietary variety was significantly associated with frailty (non-DMHV, 3.6%; non-DMLV, 6.7%; DMHV, 6.7%; DMLV, 12.2%; *p* = 0.002).

Table 3 shows the results of binomial logistic regression analyses in which the presence of frailty was used as a dependent variable. The combination of DM and a low DVS was significantly associated with frailty in the crude model, adjusted model 1, and adjusted model 2.

To examine the independent association between frailty and a low DVS or DM, another logistic regression analysis was conducted (Table 4). Both a low DVS and DM were significantly associated with frailty in adjusted models 1 and 2. When DM with hyperglycemia (HbA1c ≥ 7.0%) instead of DM was entered into the model, the significant association between a low DVS or high HbA1c and frailty was maintained (odds ratio (OR) (95% confidence interval (CI)): DM and hyperglycemia: 2.90 (1.15–7.33), low DVS: 2.20 (1.32–3.67)).

## 4. Discussion

This study indicated a significant relationship between a low DVS or DM and frailty in the older population. The prevalence of participants with DM consuming a poor dietary variety was 12.2%, which was approximately 3.4 times higher than those participants without DM consuming a high dietary variety. After adjusting for covariates, including comorbidities, the odds ratio of frailty in the group of participants with DM and a low DVS was the highest at 5.0. To the best of our knowledge, this study is the first large-scale study that examined the relationship between frailty and the combination of DM and dietary variety.

In our study, the prevalence of frailty was about twice as high in participants with DM as in those without DM. The result is consistent with that of similar cross-sectional and longitudinal studies [8,9]. This association between DM and frailty may be due to hyperglycemia (HbA1c ≥ 8.0%), hypoglycemia, low HbA1c levels, abdominal obesity, macrovascular complications, physical inactivity, or malnutrition [10,11,12,13,14]. The median HbA1c in individuals with DM in our study was approximately 6.7%, which is well-controlled. However, in a cross-sectional study of 543 people aged 70 to 79 years, frailty was 1.96 times more common in residents with an HbA1c ≥ 6.5% than in those with an HbA1c of <6.0%, suggesting that mild hyperglycemia may have some effect on frailty [29]. In contrast, the possibility that a low HbA1c or hypoglycemia, which was not assessed in our study, may have affected the increased prevalence of frailty cannot be neglected. The association between DM and frailty, after adjusting for BMI and comorbidities, suggests that lifestyle, including diet and physical activity, may have affected the increased prevalence of frailty in adults with DM. Another explanation for the association between DM and frailty could be the high prevalence of low skeletal muscle or sarcopenia in DM. Studies have reported that patients with DM have reduced muscle strength and low muscle quality [30,31]. Older individuals display a reduced muscle protein synthetic response to dietary protein/amino acids intake, termed anabolic resistance, which has a negative impact on muscle mass and function [32]. Insulin resistance causes a negative balance between protein synthesis and breakdown, which results in reduced skeletal muscle mass [33]. As we did not assess muscle mass in this study, further studies are needed to clarify whether the association between DM or DVS and frailty is mediated through sarcopenia.

In our study, a low DVS was also associated with a high prevalence of frailty. This result is consistent with another study of 665 older community residents with a mean age of 73.6 years, showing that a high DVS is associated with a low degree of frailty [21]. The association between a low DVS and frailty may be due to those with a low DVS having a lower intake of protein- or vitamin-rich foods than those with a high DVS in both the participants with and without DM. The DVS used for this study included five out of ten items comprising sources of protein, with the remaining five comprising sources of vitamins and minerals [25]. Many studies demonstrated that a low intake of protein, vitamin A, folate, vitamin D, and total energy was associated with frailty in older people [15,16,17,18]. Therefore, a high dietary variety leads to a high intake of energy, protein, and vitamins, which may contribute to the prevention of frailty [26].

In addition, a varied diet shares similarities with the Mediterranean diet and other healthy dietary patterns in that they are high in fish, vegetables, and fruits, and low in refined grains. In a meta-analysis, a high adherence to the Mediterranean or other healthy diet was associated with a reduced risk of frailty in the older population [19,20] and patients with DM [34]. It has been reported that an increase in the Mediterranean diet score by 2 points in 8970 women with type 2 DM led to a 28% reduction in the risk of frailty [34]. However, the Mediterranean foods used in the study included olive oil, nuts, and a moderate amount of wine [35], which are different from a high DVS diet in our study, which was developed based on a typical Japanese diet. A meta-analysis including non-Mediterranean countries revealed that healthy dietary patterns (a diet high in vegetables, fruit, and whole grains) reduced the risk of frailty by 30% in older people [20]. Therefore, a varied diet may provide older people with sufficient amounts of vegetables, fruits, soy, and fish, which could prevent frailty, such as with the Mediterranean diet.

In our study, we found that the combination of DM and a low DVS further exacerbates the risk of frailty. It has been reported that older adults with DM are more likely to have malnutrition compared to their counterparts without DM [36]. Excessive restriction of energy and protein, as well as the presence of DM or age-related comorbidities, could lead to weight loss, which is an early sign of frailty, as is malnutrition [37]. Low intake of protein including branched-chain amino acids (BCAA) in older diabetic patients in conjunction with anabolic resistance may further exacerbate sarcopenia and frailty through the decreased action of BCAA on insulinotropic and muscle protein synthesis [38]. The dietary strategy of older people with DM should advance with age from strict dietary restrictions to a diet aimed at preventing frailty [39]. A diet high in protein, vegetables, fruits, and fish, which has a high DVS, should be recommended in patients who are aged over 75 years or who have frailty as well as malnutrition [39]. Findings from this study suggest that a varied diet may be useful in preventing frailty in individuals with DM. Future studies are needed to examine whether the application of the DVS to clinical practice is effective in preventing frailty in older adults with DM.

This study has several limitations. First, it was a cross-sectional study, and therefore causal relationships cannot be inferred. It is necessary to conduct longitudinal and intervention studies. Second, information on DM that was collected from participants in this study was not sufficient. While a nurse interviewed participants for their past history of DM, information on the type of DM, age of onset, and medications was not collected. Information about dietary management, such as the experience of receiving nutrition counseling regarding the management of DM and the current situation of dietary control, was also unknown. Future studies would need to allocate participants to different groups according to the type of DM—that is, type 1 or type 2. Third, participants may have been healthy. The participant frailty rate was 5.5%, which was lower than that reported in earlier studies [21,27]. This may be because: (1) participants may have been highly conscious about their health as they continuously attended check-ups, or (2) participants were able to travel to the check-up venue. Lastly, the DVS is a tool used for the evaluation of the frequency of consumption of certain foods, not for the quantitative evaluation of foods consumed. Therefore, it is necessary to investigate the food and nutrient intake amounts.

## 5. Conclusions

Both a limited dietary variety and DM are independently associated with frailty in older people and the combination increased the prevalence of frailty. These findings suggest that high dietary variety could be important for the prevention of frailty in people with DM. Further studies are needed to examine whether nutritional intervention using the Dietary Variety Score is as effective as aggressive nutritional guidance targeting specific nutrients and total energy intake in improving frailty in older adults with DM.

## Figures and Tables

**Table 1:** The characteristics of participants in the 4 groups classified by the presence or absence of diabetes and a high or low Dietary Variety Score (DVS).

			Non-DMHV		Non-DMLV		DMHV		DMLV		*p*-Values
			(*n* = 644)		(*n* = 564)		(*n* = 75)		(*n* = 74)		
Age		(year)	77	72–81	76	71–80	77	73–82	75	72–80	0.158
Sex		Female	466	(72.4%)	299	(53.0%)	38	(50.7%)	29	(39.2%)	<0.001
BMI		(kg/m^2^)	22.6	20.7–24.5	23.0	21.2–25.0	22.7	21.4–25.0	24.4	22.3–27.0	<0.001
Members of household		Yes	444	(68.9%)	361	(64.0%)	49	(65.3%)	50	(67.6%)	0.336
Alcohol consumption		Yes	270	(41.9%)	292	(51.8%)	40	(53.3%)	33	(44.6%)	0.004
Smoking		Yes	34	(5.3%)	46	(8.2%)	3	(4.0%)	6	(8.1%)	0.161
MMSE		(point)	29.0	27.0–30.0	28.0	27.0–29.0	28.0	26.0–29.0	28.0	26.0–29.3	0.109
DVS		(point)	6.0	5.0–7.0	3.0	2.0–4.0	6.0	5.0–8.0	2.5	1.0–4.0	<0.001
Comorbidity											
Hypertension			273	(42.4%)	249	(44.1%)	47	(62.7%)	48	(64.9%)	<0.001
Stroke			42	(6.5%)	37	(6.6%)	2	(2.7%)	6	(8.1%)	0.544
Heart disease			108	(16.8%)	119	(21.1%)	18	(24.0%)	22	(29.7%)	0.020
Hyperlipidemia			235	(36.5%)	224	(39.7%)	43	(57.3%)	39	(52.7%)	0.001
Osteoporosis			170	(26.4%)	113	(20.0%)	9	(12.0%)	7	(9.5%)	<0.001
Spinal canal stenosis			85	(13.2%)	71	(12.6%)	6	(8.0%)	6	(8.1%)	0.393
Malignant neoplasm			113	(17.5%)	81	(14.4%)	16	(21.3%)	14	(18.9%)	0.265
Depression			13	(2.0%)	21	(3.7%)	1	(1.3%)	4	(5.4%)	0.140
Present knee pain			208	(32.3%)	187	(33.2%)	33	(44.0%)	17	(23.0%)	0.055
Laboratory test											
Serum creatinine		(mg/dL)	0.72	0.62–0.84	0.77	0.66–0.90	0.77	0.66–0.96	0.82	0.68–0.96	<0.001
Serum albumin		(g/dL)	4.2	4.0–4.4	4.2	4.1–4.4	4.2	4.1–4.5	4.2	4.1–4.4	0.373
HbA1c		(%)	5.6	5.4–5.8	5.6	5.4–5.8	6.7	6.1–7.3	6.7	6.2–7.1	<0.001

For continuous variables, the Kruskal–Wallis-test was used; for categorical variables, the chi- square test was used. non-DMHV: the no diabetes mellitus/high Dietary Variety Score group; non-DMLV: the no diabetes mellitus/low Dietary Variety Score group; DMHV, the diabetes mellitus/high Dietary Variety Score group; DMLV: the diabetes mellitus /low Dietary Variety Score group; BMI, body mass index; MMSE:Mini-Mental State Examination; DVS: Dietary Variety Score; HbA1c: hemoglobin A1c.

**Table 2:** Prevalence of robust, pre-frailty, and frailty in the 4 groups classified by the presence or absence of diabetes mellitus (DM) and a high or low DVS.

	Non-DMHV		Non-DMLV		DMHV		DMLV		*p*-Values
	**(*n* = 644)**		(*n* = 564)		(*n* = 75)		(*n* = 74)		
Frailty evaluation									
Robust	342	(53.1%)	266	(47.2%)	27	(36.0%)	30	(40.5%)	
Pre-frailty	279	(43.3%)	260	(46.1%)	43	(57.3%)	35	(47.3%)	
Frailty	23	(3.6%)	38	(6.7%)	5	(6.7%)	9	(12.2%)	0.002

The chi-square test was used. non-DMHV, the no diabetes mellitus/high Dietary Variety Score group; non-DMLV, the no diabetes mellitus/low Dietary Variety Score group; DMHV, the diabetes mellitus/high Dietary Variety Score group; DMLV, the diabetes mellitus /low Dietary Variety Score group.

**Table 3:** Binomial logistic regression analyses for the association with frailty in 4 diabetes/DVS groups in the crude model and models adjusted for covariates.

DM/DVS Group	Crude Model	Adjusted Model 1	Adjusted Model 2
DMLV	3.74 (1.66–8.42)	4.23 (1.79–10.01)	5.03 (2.05–12.35)
DMHV	1.93 (0.71–5.23)	1.81 (0.65–5.04)	1.87 (0.63–5.52)
non-DMLV	1.95 (1.15–3.32)	2.13 (1.23–3.69)	2.18 (1.25–3.83)
non-DMHV	1 (reference)	1 (reference)	1 (reference)

Adjusted model 1: adjusted for age, sex, BMI. Adjusted model 2: model 1 plus further adjustments for hypertension, hyperlipidemia, heart disease, osteoporosis, the presence of knee pain, serum creatinine, and alcohol consumption. non-DMHV, the no diabetes mellitus/high Dietary Variety Score group; non-DMLV, the no diabetes mellitus/low Dietary Variety Score group; DMHV, the diabetes mellitus/high Dietary Variety Score group; DMLV, the diabetes mellitus /low Dietary Variety Score group; DM, diabetes mellitus; DVS, Dietary Variety Score.

**Table 4:** Logistic regression analyses for the association between frailty and a low DVS or diabetes mellitus in the models adjusted for covariates.

DM/DVS Group	Adjusted Model 1	Adjusted Model 2
Low DVS	2.16 (1.31–3.57)	2.26 (1.35–3.78)
DM	1.92 (1.02–3.61)	2.13 (1.09–4.14)

Adjusted model 1: adjusted for age, sex, BMI, a low DVS, and diabetes. Adjusted model 2: model 1 plus further adjustments for hypertension, hyperlipidemia, heart disease, osteoporosis, the presence of knee pain, serum creatinine, and alcohol consumption. non-DMHV, the no diabetes mellitus/high Dietary Variety Score group; non-DMLV, the no diabetes mellitus/low Dietary Variety Score group; DMHV, the diabetes mellitus/high Dietary Variety Score group; DMLV, the diabetes mellitus /low Dietary Variety Score group; DM, diabetes mellitus; DVS, Dietary Variety Score.

## Data Availability

The data presented in this study are available on request from the corresponding author. The data are not publicly available due to ethicolegal restrictions imposed by the Ethics Committee at Tokyo Metropolitan Institute of Gerontology.

## Supplementary Materials

The following are available online at https://www.mdpi.com/2072-6643/13/2/641/s1, Table S1: Presence of diabetes and DVS.
