# Supplementary material for: Low Dietary Variety and Diabetes Mellitus Are Associated with Frailty among Community-Dwelling Older Japanese Adults: A Cross-Sectional Study
Source: Nutrients. 2021 Feb 16;13(2):641. doi: 10.3390/nu13020641 (PMC7920314; doi:10.3390/nu13020641)
Supplement: Supplementary file 1 [file nutrients-13-00641-s001.pdf]

Supplemental data 1: The frequency of all foods consumed daily between the high DVS and low DVS groups in participants with and without diabetes mellitus

	non-DMHV (n=644)	non-DMLV (n=564)	p-values	DMHV (n=75)	DMLV (n=74)	p-values
Seafood	363 (56.4%)	78 (13.8%)	<0.001	40 (53.3%)	15 (20.3%)	<0.001
Meat	372 (57.8%)	82 (14.5%)	<0.001	42 (56.0%)	5 (6.8%)	<0.001
Eggs	430 (66.8%)	149 (26.4%)	<0.001	54 (72.0%)	22 (29.7%)	<0.001
Milk	484 (75.2%)	235 (41.7%)	<0.001	52 (69.3%)	29 (39.2%)	<0.001
Soy	494 (76.7%)	189 (33.5%)	<0.001	62 (82.7%)	28 (37.8%)	<0.001
Green and yellow vegetable	566 (87.9%)	246 (43.6%)	<0.001	67 (89.3%)	32 (43.2%)	<0.001
Seaweed	356 (55.3%)	52 (9.2%)	<0.001	44 (58.7%)	5 (6.8%)	<0.001
Potato	174 (27.0%)	18 (3.2%)	<0.001	20 (26.7%)	1 (1.4%)	<0.001
Fruit	539 (83.7%)	288 (51.1%)	<0.001	59 (78.7%)	29 (39.2%)	<0.001
Fats and oils	492 (76.4%)	186 (33.0%)	<0.001	51 (68.0%)	14 (18.9%)	<0.001

The chi-square test was used.
